# Impact of standardization on racial and socioeconomic disparities in non-accidental trauma evaluations in infants in a pediatric emergency department

**DOI:** 10.1186/s40621-023-00441-w

**Published:** 2023-07-03

**Authors:** Laura Even Elliott, Michael A. Gittelman, Eileen M. Kurowski, Elena M. Duma, Wendy J. Pomerantz

**Affiliations:** 1 Division of Emergency Medicine, Cincinnati Children’s Hospital, 3333 Burnet Avenue, ML #1005, Cincinnati, OH 45229 USA; 2 Division of Emergency Medicine, Comprehensive Children’s Injury Center, Cincinnati Children’s Hospital, 3333 Burnet Avenue, ML #2008, Cincinnati, OH 45229 USA; 3 Division of Emergency Medicine, James M. Anderson Center for Health Systems Excellence, Cincinnati Children’s Hospital, 3333 Burnet Avenue, ML #7014, Cincinnati, OH 45229 USA; 4 Division of Emergency Medicine, Cincinnati Children’s Hospital, 3333 Burnet Avenue, ML #2008, Cincinnati, OH 45229 USA

**Keywords:** Non-accidental trauma, Infant, Standardization, Disparities

## Abstract

**Background:**

Studies have illustrated racial and socioeconomic disparities in evaluation of non-accidental trauma (NAT). We aimed to investigate how implementation of a standardized NAT guideline in a pediatric emergency department (PED) impacted racial and socioeconomic disparities in NAT evaluation.

**Results:**

1199 patients (541 pre- and 658 post-guideline) were included for analysis. Pre-guideline, patients with governmental insurance were more likely than those with commercial insurance to have a social work (SW) consult completed (57.4% vs. 34.7%, *p* < 0.001) and a Child Protective Services (CPS) report filed (33.4% vs. 13.8%, *p* < 0.001). Post-guideline, these disparities were still present. There were no differences in race, ethnicity, insurance type, or social deprivation index (SDI) in rates of complete NAT evaluations pre- or post-guideline implementation. Overall adherence to all guideline elements increased from 19.0% before guideline implementation to 53.2% after (*p* < 0.001).

**Conclusion:**

Implementation of a standardized NAT guideline led to significant increase in complete NAT evaluations. Guideline implementation was not associated with elimination of pre-existing disparities in SW consults or CPS reporting between insurance groups.

## Background

Multiple studies have shown disparities in the evaluation of non-accidental trauma (NAT) in both the pediatric emergency department (PED) and inpatient settings. Non-White patients with injuries concerning for NAT are more likely to have skeletal surveys obtained (Hymel et al. 2018; Lane et al. 2002; Wood et al. 2010). Publicly or uninsured patients have a higher relative risk of a Child Protective Services (CPS) report being filed compared to privately insured patients (Lane et al. 2002). A similar pattern was seen in a national database review of infants with traumatic brain injuries, in which publicly or uninsured patients were more likely to receive skeletal surveys and be diagnosed with child abuse (Wood et al. 2010).

The impact of standardization on racial/ethnic and socioeconomic disparities of NAT evaluations has been promising, though understudied. A retrospective study showed that for pediatric patients admitted to the hospital with unwitnessed head trauma, a standardized screening algorithm eliminated racial disparities in ordering skeletal surveys (Rangel et al. 2009). In a study investigating patients less than 12 months old with skeletal fractures, implementation of a guideline eliminated the increased likelihood of children with government-subsidized or no insurance to have skeletal surveys completed (Higginbotham et al. 2014). One multi-center study showed the opposite effect of guideline implementation, with presence of a guideline being associated with increased disparities between patients with private and public insurance (Stavas et al. 2020).

In 2015, Cincinnati Children’s Hospital Medical Center’s (CCHMC) PED implemented a standardized age-based clinical care guideline for evaluation of patients presenting with injuries concerning for NAT. The guideline was developed by a team of PED physicians and child abuse specialists utilizing both peer-reviewed literature and expert consensus. The guideline lists injuries that should raise concern for NAT based on whether a patient is ambulatory or pre-ambulatory. In pre-ambulatory patients, which this study focused on, injuries that should raise concern for NAT include bruise (Harper et al. 2014) , burn (Degraw et al. 2010), laceration, mouth injury such as frenulum tear (Maguire et al. 2007), eye injury including subconjunctival hemorrhage (DeRidder et al. 2013), intracranial injury (John et al. 2013), abdominal injury (Wood et al. 2005), genital injury, and any fracture (Leventhal et al. 2010). In ambulatory patients, NAT should be considered if patients have bruises in non-bony locations, patterned injuries or those in multiple stages of healing, or if there is a concerning or inconsistent history.

The recommended workup is then divided by age: < 6 months, 6–12 months, > 12–36 months. All patients under 36 months presenting with injuries concerning for NAT should receive a social work consult and a full exam. Because patients under 6 months are pre-ambulatory, the guideline recommends that when these patients present with one of the listed injuries, they should also receive a skeletal survey, head computed tomography (CT), aspartate transaminase (AST), alanine transaminase (ALT), and lipase. Additional labs are recommended for specific injuries and based on results of the initial testing, but these are not uniformly recommended in all NAT evaluations.

Guideline introduction in the PED was paired with a quality improvement (QI) initiative. This process included provider education, a copy of the guideline in the electronic health record (EHR) “E-brain,” and introduction of order sets in the EHR. With the new guideline and the associated QI project, percentage of guideline-adherent NAT evaluations rose from 47 to 68.5% across all age groups, and adherence reached steady state in 2016 (Riney et al. 2018). To our knowledge, no study has looked at disparities in complete guideline adherence for NAT evaluation in children less than 6 months of age, with specific injuries that can be seen in NAT, before and after guideline implementation.

We focused on children under 6 months of age primarily because these patients are pre-ambulatory. Therefore there are specific injuries in these children that should trigger a provider to initiate the NAT workup. Children in the older age groups may or may not be ambulatory, and many of the injuries that should trigger an NAT evaluation in these children are conditional on location and history, making these groups more difficult to study.

The primary aim of this study was to describe patients under 6 months of age presenting with complaints associated with NAT and to determine the proportion undergoing guideline-adherent evaluation pre- and post-guideline implementation. Additionally, we evaluated disparities in completion of NAT evaluation pre- and post-guideline implementation to assess if there were any racial, ethnic, or socioeconomic disparities.

## Results

**Fig. 1:**
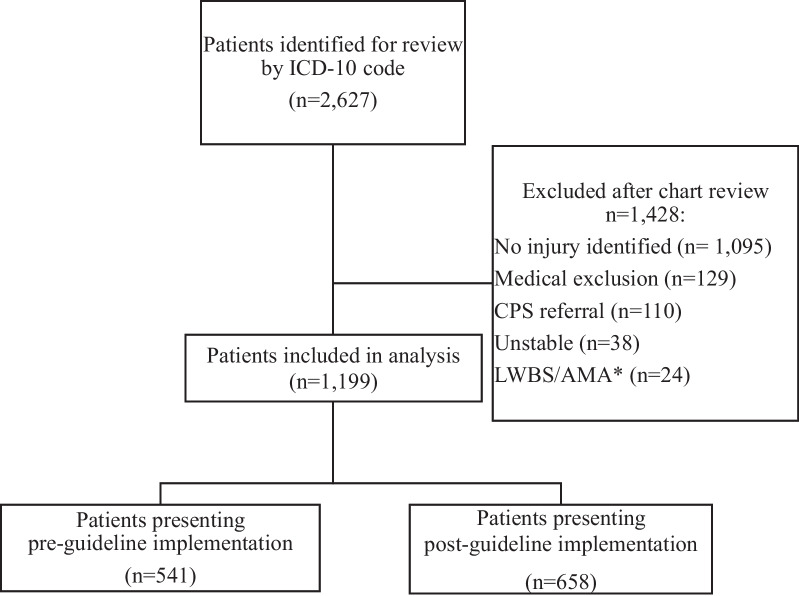
Study flow diagram. *LWBS/AMA = Left without being seen/against medical advice

Figure 1 shows the study flow diagram. Based on age, date of presentation, and ICD-10 code, 2627 patients were identified as eligible for review via automated extraction from the EHR. A total of 1199 patients were included in analysis, with 541 (45.1%) patients in the pre-guideline group and 658 (54.8%) in the post-guideline group. There were 53 addresses that could not be geocoded and 29 patients with addresses listed at the Job and Family Services; these patients were not included in the social deprivation index (SDI) analysis

Table 1 shows the demographics of the pre- and post-guideline groups. The only statistically significant differences between the two groups were that the post-guideline average age was 10 days younger than the pre-guideline average age, and there was an increase in the proportion of patients with governmental insurance post-guideline implementation.

**Table 1:** Demographics of pre- and post-guideline groups

	Pre-guideline *n* = 541	Post-guideline *n* = 658	*p* value
** Mean age (days)**	103	93	0.001
** Male sex, n (%)**	295 (54.5)	352 (53.5)	0.721
** Race**, **n (%)**			
White	358 (66.2)	441 (67.0)	0.757
Black/African American	113 (20.9)	142 (21.6)	
Multiracial	33 (6.1)	31 (4.7)	
**Ethnicity**, **n (%)**			
Non-Hispanic	512 (94.6)	628 (95.4)	0.164
Hispanic	17 (3.1)	24 (3.6)	
** Insurance type**, **n (%)**			
Governmental	338 (62.5)	431 (65.5)	0.028
Commercial	196 (36.2)	206 (31.3)	
Self-pay/other/unknown	7 (1.3)	21 (3.2)	
Mean social deprivation index (SD)*	0.341 (0.145)	0.343 (0.138)	0.829

*n = 510 pre-guideline and *n* = 607 post-guideline

*SD* standard deviation

The mean length of stay in the PED increased from 270 min pre-guideline to 295 min post-guideline implementation. In both groups, fractures were the most common injury type (37.0% pre-guideline, 36.2% post-guideline), followed by bruises (23.7% pre-guideline, 20.5% post-guideline). There were no differences in presentation location (main campus vs. satellite), triage level, or disposition between the pre- and post-guideline groups.

Table 2 shows the compliance with individual components of the guideline as well as complete guideline adherence pre- and post-guideline implementation. Evaluation completion was higher in the post-guideline group across all components. The rate of CPS reporting was also higher.

**Table 2:** Guideline compliance pre- and post-guideline implementation

	Pre-guideline, *n* (%)	Post-guideline, *n* (%)	*p* value
Skeletal survey	272 (50.3)	417 (63.4)	< 0.001
Head CT	340 (62.8)	483 (73.4)	< 0.001
All labs	134 (24.8)	389 (59.1)	< 0.001
SW consult	265 (49.0)	442 (67.2)	< 0.001
Complete evaluation	103 (19.0)	350 (53.2)	< 0.001
CPS report filed	141 (26.1)	211 (32.1)	0.023
Complete evaluation and CPS report filed	69 (12.8)	184 (28.0)	< 0.001

Complete evaluation was defined as skeletal survey, head CT, AST, ALT, lipase, and a social work consult. *SW* social work, *CPS* Child Protective Services

Pre-guideline implementation, patients with governmental insurance were more likely than those with commercial insurance to have a SW consult (57.4% vs. 34.7%, *p* < 0.001) and a CPS report filed (33.4% vs. 13.8%, *p* < 0.001). Post-guideline implementation, patients with governmental insurance were still more likely than those with commercial insurance to have a SW consult completed (71.0% vs. 59.2%, *p* < 0.001) and a CPS report filed (38.7% vs. 16.5%, *p* < 0.001). There were no statistically significant differences in complete evaluation among racial, ethnic, insurance, or social deprivation groups. The disparities in SW consult and CPS reports seen between insurance types were not seen among racial or ethnic groups pre- or post-guideline.

## Discussion

This study is one of few studies to investigate racial, ethnic, and socioeconomic disparities in NAT evaluation in a PED. It is the first study to evaluate how standardization in NAT evaluation impacts disparities in all aspects of NAT evaluation, including laboratory testing, diagnostic imaging, SW consults, and CPS reporting. Both pre- and post-guideline implementation, patients with governmental insurance were more likely to have SW consults placed and CPS reports made. The difference in the rate of SW consults was less in the post-guideline group; before implementation, 25% more patients with governmental insurance had a SW consult compared to those with commercial insurance, and after implementation, only 12% more patients with governmental insurance had a SW consult. No racial, ethnic, or SDI disparities were seen in SW consults or CPS reporting pre- or post-guideline implementation. No racial, ethnic, SDI, or insurance disparities were seen in complete evaluation pre- or post-guideline implementation.

Our study illustrated a disparity in NAT evaluation; specifically, patients with governmental insurance had higher rates of SW consults and CPS reporting. This is consistent with prior studies showing similar disparities in CPS reporting among insurance groups (Lane et al. 2002; Rebbe et al. 2022). Our study did not illustrate the racial or ethnic disparities in skeletal surveys or CPS reporting seen in prior studies (Hymel et al. 2018; Lane et al. 2002; Wood et al. 2010).

Guideline implementation was associated with an increase in guideline-adherent NAT evaluation with adherence rising from 19 to 53%. A prior QI study in our ED illustrated a rise in guideline adherence, from 47 to 68.5%; however, the previous study included all age groups and did not include the non-specific injuries that required manual chart review (Riney et al. 2018). Our study’s adherence rates for skeletal survey and SW consult are also lower than those demonstrated by Higginbotham et al. (2014). Their study specifically focused on skeletal fractures in patients under 12 months, though the average age of patients included was greater than 6 months of age.

Our study showed that implementation of NAT guideline was not associated with an elimination of pre-existing disparities in SW consults or CPS reporting among insurance groups. However, the difference in the rate of SW consults was smaller in the post-guideline group: before implementation, 25% more patients with governmental insurance had a SW consult compared to those with commercial insurance, and after implementation, only 12% more patients with governmental insurance had a SW consult. This is similar to the findings of Higginbotham et al. (2014). A previous study suggested an association between guideline implementation and insurance disparities in skeletal surveys (Stavas et al. 2020), and our study suggests that guideline implementation is in fact not a cause for disparities but rather there may be another factor contributing to both guideline implementation and disparities.

Our study has limitations. As a retrospective chart review, our data are dependent on what is documented by the provider at the time of the PED visit; however, there is no reason that documentation would have been systematically different pre- and post-guideline implementation. There is also the subjectivity of chart review; however, 20 charts were re-reviewed by the principal investigator to try to ensure validity and reliability of the review. Despite provider education and an order set in the EHR, compliance with the guideline was still relatively low at 53% within our population. Race is a social construct; its use in this study was as a social marker and a potential risk for bias and not as a biologic proxy. Finally, while our guideline was implemented in 2015, other factors in our healthcare system and more globally may have also impacted changes in NAT evaluation in our PED.

## Conclusions

In our PED, implementation of a standardized NAT guideline led to significant increase in complete NAT evaluations. Guideline implementation was not associated with elimination of pre-existing disparities in SW consults or CPS reporting among insurance groups and did not introduce new racial or ethnic disparities in NAT evaluations. Despite provider education and an order set in the EHR, compliance with the guideline was still relatively low at 53% within our population. Further study will be needed to determine whether increased testing leads to increased injury identification as well as why, despite guidelines and education, adherence to NAT guideline remains relatively low.

## Methods

### Data source

This was a retrospective chart review of all patients aged less than 6 months, all of whom are pre-ambulatory, who presented with an injury diagnosis concerning for NAT, seen at a large pediatric tertiary care level 1 trauma center PED and its satellite community PED, between January 1, 2012 and December 31, 2020. The guideline was implemented in 2015, and a steady state of guideline adherence was reached in January 2016 (Riney et al. 2018). Data were obtained electronically from EPIC, the EHR, as well as manual chart review. This study was approved as exempt by the institutional review board at CCHMC (IRB 2021−0211).

### Study population

Based on the NAT guideline, patients were included if they were less than 6 months old and had an ICD-10 code for one or more of the following injury types or mechanisms: bruise/contusion, burn, laceration, mouth injury including frenulum tear or lip laceration, eye injury including subconjunctival hemorrhage or retinal hemorrhage, head injury, intracranial injury, abdominal injury, genital injury, fracture, gunshot wound. Additionally, we studied those with ICD-10 codes as follows: alleged physical abuse, suspected child physical abuse, rule out physical abuse, non-accidental physical abuse, non-accidental traumatic injury to child and personal history of physical abuse. Patients were excluded if their injuries were consistent with documented birth trauma or cephalohematomas/subconjunctival hemorrhages in the first 2 weeks of life or recent documented surgical or medical treatment. Patients were also excluded if there was documentation that the injury event was observed and confirmed by an impartial witness in a public location, if the injury was the result of a motor vehicle crash, or if the injury was an animal bite, hair tourniquet, or corneal abrasion. Additionally, patients critically unstable upon arrival (prohibiting completion of full NAT evaluation in the PED), patients with known medical problems that increase risk of injuries including metabolic bone disease and coagulation/bleeding disorders, as well as patients referred by CPS because their siblings were seen for known abuse were excluded. Patients presenting with a chief complaint describing an injury but with no visible injury on documented physical exam were also excluded. For patients presenting with the same injury multiple times, only the first encounter was included for analysis.

A steady state of guideline adherence was reached in January 2016 (Riney et al. 2018), therefore patients presenting between January 1, 2012 and December 31, 2015 were categorized as the “pre-guideline” group, while patients presenting between January 1, 2016 and December 31, 2020 were categorized as “post-guideline” group.

### Data set

Variables abstracted directly from the EHR included: patient demographics (insurance type, address of residence, race, ethnicity, age), triage information, date and time of arrival, arrival location, PED disposition, admitting unit if admitted, medical/injury history, standard abuse evaluation components obtained (skeletal survey, head CT, ALT, AST, lipase), injury data including type of injury, mechanism of injury, intent of injury, and location of injury. In our institutional guideline, additional laboratory studies are recommended depending on the results of the initial studies and the location of a child’s injury. Because these are not universally recommended, we did not include them in our definition of a complete workup. Manual review of all charts followed a standardized chart review process. All chart review was conducted by the clinician investigators, and uncertainties were discussed as a group until consensus was reached. The first 20 charts reviewed by each investigator were co-reviewed by the first author to ensure accurate and reliable coding. Variables manually abstracted from the EHR included previous medical problems, previous visits for injuries, whether a SW consult was obtained, and whether a report to CPS was filed. All ICD-10 codes were reviewed for each patient, and encounters with diagnoses that clearly met exclusion criteria were eliminated. Charts for patients with ICD-10 codes for unspecified injuries of the mouth, eye, head, abdomen, extremities, genitals, or unspecified location of injury were manually reviewed by the study investigators to determine eligibility for inclusion.

Patient charts with missing demographic data that precluded categorization into race or socioeconomic groups were grouped together in an “unknown” category. Ethnicity and race data were categorized and coded for analysis. Patients had the ability to self-report up to two race categories. For analysis, patients were categorized into the following race categories: White, Black/African American, Multiracial Black/White, and Other/unknown. Patients could select Hispanic or non-Hispanic ethnicity. If Hispanic/Latino was listed as a race, the patient’s ethnicity was identified as “Hispanic,” and their race was categorized by the other listed race or “unknown” if a second race was not selected.

All patients’ home addresses were geocoded to a specific census tract, and a SDI was assigned. SDI is a score that is calculated from neighborhood-level socioeconomic variables obtained from the 2011–2015 U.S. Census American Community Survey. SDI is based on 8 different socioeconomic measures to quantify the degree of deprivation for each census tract and ranges from 0 (low deprivation) to 1 (high deprivation) (Brokamp et al. 2016). Patients with addresses that could not be geocoded (*n* = 53) and those in the custody of CPS at the time of evaluation (*n* = 29) were excluded from the SDI analysis. For those in CPS custody, their listed address was the address of the county Job and Family Services, precluding categorization into address-based socioeconomic group.

### Statistical analysis

Descriptive statistics were used to characterize the population. Chi-Square test and student’s t-tests were used to assess differences between groups for categorical and continuous variables, respectively. Statistical significance was defined as p < 0.05. Data were analyzed with IBM® SPSS® Statistics, Version 26.0.

## Data Availability

The datasets used and/or analyzed during the current study are available from the corresponding author on reasonable request.

## Abbreviations

ALT: Alanine transaminase
AMA: Against medical advice
AST: Aspartate transaminase
CCHMC: Cincinnati Children’s Hospital Medical Center
CPS: Child Protective Services
CT: Computed tomography
EHR: Electronic health record
LWBS: Left without being seen
NAT: Non-accidental trauma
PED: Pediatric emergency department
QI: Quality improvement
SDI: Social deprivation index
SW: Social work
